# Supplementary material for: A systematic review and meta-analysis on international studies of prevalence, mortality and survival due to coal mine dust lung disease
Source: PLoS One. 2021 Aug 3;16(8):e0255617. doi: 10.1371/journal.pone.0255617 (PMC8330946; doi:10.1371/journal.pone.0255617)
Supplement: S1 Table — (PDF) [file pone.0255617.s002.pdf]

S1 File Search strategies

The electronic databases PUBMED and EMBASE (2000 –15/02/2021) were searched for English-language academic peer-reviewed articles.

Search queries were conducted in a stepwise manner by breaking down each topic (e.g. coal mine dust lung diseases or prevalence or mortality or survival) into key concepts (each numbered step in Table 2) and searching individually. For each topic, alternative terms were used to cover all possible synonyms for that component. Individual search queries were then combined to create the final search query using BOOLEAN operators such as “AND” or “OR”. Filters used were: Publication date from 2000/01/01 to 2021/12/31; Humans; English.

1.1 PUBMED

Search	Query
#1	Search ((((((("coal mining"[MeSH Terms]) OR "coal mining"[Text Word]) OR "coal miners"[Text Word]) OR "surface coalminers"[Text Word]) OR "underground coal miners"[Text Word])) AND ((((((((((((((("pneumoconiosis"[MeSH Terms] OR "pneumoconiosis/epidemiology"[MeSH Terms]))) OR (("black lung disease"[Text Word] OR "black lung diseases"[Text Word]))) OR (("coal worker pneumoconiosis"[Text Word] OR "coal worker s lung"[Text Word] OR "coal worker s pneumoconiosis"[Text Word] OR "coal worker s pneumoconiosis"[Text Word] OR "coal worker's lung"[Text Word] OR "coal worker's pneumoconiosis"[Text Word] OR "coal worker's pneumoconiosis"[Text Word] OR "coal workers lung disease"[Text Word] OR "coal workers pneumoconiosis"[Text Word]))) OR (("coal miners lung"[Text Word] OR "coal miners lungs"[Text Word] OR "coal miners pneumoconiosis"[Text Word] OR "coal miners' pneumoconiosis"[Text Word] OR "coal miners, pneumoconiosis"[Text Word]))) OR "Coal Mine dust lung disease"[Text Word]) OR "anthracosis"[Text Word]) OR "anthracosis"[MeSH Terms]) OR "silicosis"[MeSH Terms]) OR (("silicosis"[Text Word] OR "silicosis/pneumoconiosis"[Text Word] OR "silicosis/pneumoconiosis"[Text Word]))) OR "dust related diffuse fibrosis"[Text Word]) OR "progressive massive fibrosis"[Text Word]) OR "grinders disease"[Text Word]) OR "mixed lung pneumoconiosis"[Text Word]) OR (("chronic obstructive pulmonary disease"[Text Word] OR "chronic obstructive pulmonary disease/copd"[Text Word]))) OR "pulmonary disease, chronic obstructive/epidemiology"[MeSH Terms]))
#2	(((“prevalence”[MeSH Terms]) OR prevalence [Title]) OR prevalence [Abstract]) OR (((("survival"[MeSH Terms]) OR survival[Title]) OR survival[Abstract]) OR "survival analysis "[MeSH Terms]))
#3	#1 AND #2 Filters: Publication date from 2000/01/01 to 2021/12/31; Humans; English

1.2 EMBASE

Search	Query
#1	((('coal'/exp OR coal) AND ('mining'/exp OR mining)) OR (('coal'/de OR coal) AND ('miners'/de OR miners))) AND ((black AND lung AND disease) OR ('coal worker pneumoconiosis'/exp OR 'coal worker pneumoconiosis') OR 'silicosis' OR ('grinders disease' OR 'progressive massive fibrosis'/exp OR 'progressive massive fibrosis' OR 'silicosis'/exp OR 'silicosis' OR 'chronic obstructive lung disease'/exp OR 'chronic obstructive lung disease')))
#2	'prevalence'/exp OR 'prevalence'
#3	'survival'/exp OR 'survival'
#4	(('prevalence'/exp OR 'prevalence') AND ((('coal'/exp OR coal) AND ('mining'/exp OR mining)) OR (('coal'/de OR coal) AND ('miners'/de OR miners))) AND ((black AND lung AND disease) OR ('coal worker pneumoconiosis'/exp OR 'coal worker pneumoconiosis') OR 'silicosis' OR ('grinders disease' OR 'progressive massive fibrosis'/exp OR 'progressive massive fibrosis' OR 'silicosis'/exp OR 'silicosis' OR 'chronic obstructive lung disease'/exp OR 'chronic obstructive lung disease'))) AND ([article]/lim OR [article in press]/lim) AND [english]/lim AND [2008-2021]/py
#5	(('survival'/exp OR 'survival') AND ((('coal'/exp OR coal) AND ('mining'/exp OR mining)) OR (('coal'/de OR coal) AND ('miners'/de OR miners))) AND ((black AND lung AND disease) OR ('coal worker pneumoconiosis'/exp OR 'coal worker pneumoconiosis') OR 'silicosis' OR ('grinders disease' OR 'progressive massive fibrosis'/exp OR 'progressive massive fibrosis' OR 'silicosis'/exp OR 'silicosis' OR 'chronic obstructive lung disease'/exp OR 'chronic obstructive lung disease'))) AND ([article]/lim OR [article in press]/lim) AND [english]/lim AND [2008-2021]/py
