# Supplementary material for: A systematic review and meta-analysis on international studies of prevalence, mortality and survival due to coal mine dust lung disease
Source: PLoS One. 2021 Aug 3;16(8):e0255617. doi: 10.1371/journal.pone.0255617 (PMC8330946; doi:10.1371/journal.pone.0255617)
Supplement: S2 Table — (PDF) [file pone.0255617.s003.pdf]

S2 Table: Critical Appraisal Tools

The Joanna Briggs Institute (JBI) Critical Appraisal Checklist for cohort studies

Major Components	Response options			
1. Were the two groups similar and recruited from the same population?	Yes	No	Unclear	Not applicable
2. Were the exposures measured similarly to assign people to both exposed and unexposed groups?	Yes	No	Unclear	Not applicable
3. Was the exposure measured in a valid and reliable way?	Yes	No	Unclear	Not applicable
4. Were confounding factors identified?	Yes	No	Unclear	Not applicable
5. Were strategies to deal with confounding factors stated?	Yes	No	Unclear	Not applicable
6. Were the groups/participants free of the outcome at the start of the study (or at the moment of exposure)?	Yes	No	Unclear	Not applicable
7. Were the outcomes measured in a valid and reliable way?	Yes	No	Unclear	Not applicable
8. Was the follow up time reported and sufficient to be long enough for outcomes to occur?	Yes	No	Unclear	Not applicable
9. Was follow up complete, and if not, were the reasons to loss to follow up described and explored?	Yes	No	Unclear	Not applicable
10. Were strategies to address incomplete follow up utilized?	Yes	No	Unclear	Not applicable
11. Was appropriate statistical analysis used?	Yes	No	Unclear	Not applicable

The Joanna Briggs Institute (JBI) Critical Appraisal Checklist for studies reporting prevalence data

Major Components	Response options			
1. Was the sample frame appropriate to address the target population?	Yes	No	Unclear	Not applicable
2. Were study participants sampled in an appropriate way?	Yes	No	Unclear	Not applicable
3. Was the sample size adequate?	Yes	No	Unclear	Not applicable
4. Were the study subjects and the setting described in detail?	Yes	No	Unclear	Not applicable
5. Was the data analysis conducted with sufficient coverage of the identified sample?	Yes	No	Unclear	Not applicable
6. Were valid methods used for the identification of the condition?	Yes	No	Unclear	Not applicable
7. Was the condition measured in a standard, reliable way for all participants?	Yes	No	Unclear	Not applicable
8. Was there appropriate statistical analysis?	Yes	No	Unclear	Not applicable
9. Was the response rate adequate, and if not, was the low response rate managed appropriately?	Yes	No	Unclear	Not applicable
