# Supplementary material for: A systematic review and meta-analysis on international studies of prevalence, mortality and survival due to coal mine dust lung disease
Source: PLoS One. 2021 Aug 3;16(8):e0255617. doi: 10.1371/journal.pone.0255617 (PMC8330946; doi:10.1371/journal.pone.0255617)
Supplement: S3 Table — (PDF) [file pone.0255617.s004.pdf]

S3 Table: Results of Critical appraisal for studies included in systematic review

S3a: Prevalence studies

	Critical appraisal questions [a]									Risk of bias [b,c]	
**Author, year**	Q1	Q2	Q3	Q4	Q5	Q6	Q7	Q8	Q9	**Total score**	**Category**
Almberg et al. 2018 [1]	N	Y	N	Y	Y	Y	Y	Y	N/A	6	Moderate
Antao et al. 2005 [2]	N	Y	Y	Y	Y	Y	Y	Y	N/A	7	Low
Arif et al. 2020 [3]	N	Y	Y	Y	Y	Y	Y	Y	N/A	7	Low
Blackley et al. 2014 [4]	N	Y	Y	Y	Y	Y	Y	Y	N/A	7	Low
Blackley et al. 2018a [5]	N	Y	U	Y	Y	Y	Y	Y	N/A	6	Moderate
Blackley et al. 2018b [6]	N	N/A	Y	Y	Y	Y	Y	Y	N/A	6	Moderate
CDC 2000 [7]	N	Y	N	Y	Y	Y	Y	Y	N/A	6	Moderate
CDC 2003 [8]	N	Y	Y	Y	Y	Y	Y	Y	N/A	7	Low
CDC 2006 [9]	N	Y	U	Y	Y	Y	Y	Y	N/A	6	Moderate
CDC 2007 [10]	N	Y	N	Y	Y	Y	Y	Y	N/A	6	Moderate
CDC 2012 [11]	N	Y	N	Y	Y	Y	Y	Y	N/A	6	Moderate
Graber et al. 2012 [12]	N	Y	N	Y	N	Y	Y	Y	N	6	Moderate
Graber et al. 2017 [13]	N	Y	N	Y	N	Y	Y	Y	N/A	5	Moderate
Hall et al. 2019 [14]	N	Y	Y	Y	Y	Y	Y	Y	N/A	7	Low
Hall et al. 2020 [15]	N	Y	N	Y	Y	Y	Y	Y	N/A	6	Moderate
Han et al. 2015 [16]	N	U	N	Y	U	Y	Y	Y	U	4	High
Han et al. 2016 [17]	N	U	Y	Y	U	Y	Y	Y	Y	6	Moderate
Kurth et al. 2020 [18]	N	Y	N	Y	Y	Y	Y	Y	N/A	6	Moderate
Laney & Atfield 2010. [19]	N	Y	Y	Y	Y	Y	Y	Y	N/A	7	Low
Laney & Atfield 2014. [20]	N	Y	U	Y	Y	Y	Y	Y	N/A	6	Moderate
Laney et al. 2010 [21]	N	Y	Y	Y	Y	Y	Y	Y	N/A	7	Low
Laney et al. 2012 [22]	N	Y	N	Y	Y	Y	Y	Y	N/A	6	Moderate
Laney et al. 2017 [23]	N	Y	Y	Y	Y	Y	Y	Y	N/A	7	Low
Li et al. 2018 [24]	N	U	N	Y	N	Y	Y	Y	U	4	High
Naidoo et al. 2004 [25]	N	Y	U	Y	U	Y	Y	Y	U	6	Moderate
Reynolds et al. 2017 [26]	N	Y	N	Y	Y	Y	Y	Y	N/A	6	Moderate
Scarisbrick et al. 2002 [27]	N	Y	Y	Y	U	Y	Y	Y	U	6	Moderate
Suarthana et al. 2011 [28]	N	Y	Y	Y	Y	Y	Y	Y	N/A	7	Low
Tor et al. 2010 [29]	N	U	U	Y	U	Y	Y	Y	U	4	High
Vallyathan et al. 2011 [30]	N	N/A	U	Y	N/A	Y	Y	Y	N/A	4	High
Wang et al. 2013 [31]	N	Y	N	Y	Y	Y	Y	Y	N/A	6	Moderate

Y=yes; N=no; U=unclear; N/A=not applicable.

a. Q. 1: Sample frame appropriate, Q. 2: Participants were appropriately sampled, Q. 3: Sample size was adequate, Q. 4: Study subjects and the setting were described, Q. 5: Data analysis was conducted with sufficient coverage of sample, Q. 6: Valid methods used to identify condition of interest, Q. 7 Condition measured in standard reliable way for all participants, Q. 8 Appropriate statistical analyses were used, Q. 9 was response rate adequate
b. Risk of bias based on Joanna Briggs Institute critical appraisal tools for cohort studies.
c. Risk of bias ranked as low risk of bias if at least 70% of all answers were yes ($7 \leq score \leq 9$), moderate risk if 50 to 69% questions were yes ($5 \leq score \leq 6$) and high risk of bias if yes answers were below 50% ($0 \leq score \leq 4$).

S3b: Mortality/Survival studies

	Critical appraisal questions [a]											Risk of bias [b,c]	
**Author, year**	Q1	Q2	Q3	Q4	Q5	Q6	Q7	Q8	Q9	Q10	Q11	**Total score**	**Category**
	Mortality												
Attfield & Kuempel 2008. [32]	Y	Y	Y	N/A	N/A	Y	Y	Y	Y	N/A	Y	8	Low
Beggs et al. 2015 [33]	Y	Y	Y	N/A	N/A	Y	Y	Y	Y	N/A	Y	8	Low
Bell & Mazurek. 2020 [34]	Y	Y	Y	N/A	N/A	Y	Y	Y	Y	N/A	Y	8	Low
CDC 2010 [35]	Y	Y	Y	N/A	N/A	Y	Y	Y	Y	N/A	Y	8	Low
Coggon et al. 2010 [36]	U	Y	Y	N/A	N/A	Y	Y	Y	Y	N/A	Y	7	Moderate
Graber et al 2014. [37]	U	Y	Y	Y	Y	Y	Y	Y	Y	N/A	Y	9	Low
Miller & MacCalman. 2010 [38]	Y	Y	Y	N/A	N/A	Y	Y	Y	Y	N/A	Y	8	Low
Smith et al. 2006 [39]	Y	Y	Y	N/A	N/A	Y	Y	Y	Y	N/A	Y	8	Low
	Survival												
Han et al. 2017 [40]	N	Y	N	Y	Y	Y	Y	U	U	N	N	5	High

Y=yes; N=no; U=unclear; N/A=not applicable.

a. Q. 1: Participants were appropriately sampled, Q. 2: Exposure clearly defined and described, Q. 3: Exposure measured in a valid and reliable way, Q. 4: Confounding factors identified, Q. 5: Confounding factors accounted for, Q. 6: Participants free of outcome at start of study, Q. 7: Outcome measured in a valid and reliable way, Q. 8: Follow up reported and sufficient, Q. 9: Follow up complete or reasons for loss described, Q. 10: Strategies to address incomplete follow up, Q. 11: Appropriate statistical analyses were used
b. Risk of bias based on Joanna Briggs Institute critical appraisal tools for cohort studies.
c. Risk of bias ranked as low risk of bias if at least 70% of all answers were yes (8≤ score ≤11), moderate risk if 50 to 69% questions were yes (:6 ≤ score ≤7) and high risk of bias if yes answers were below 50% (0 ≤ score ≤5).

References

1. Almberg KS, Halldin CN, Blackley DJ, Laney AS, Storey E, Rose CS, et al. Progressive Massive Fibrosis Resurgence Identified in U.S. Coal Miners Filing for Black Lung Benefits, 1970-2016. Ann Am Thorac Soc. 2018;15(12):1420-6. doi: 10.1513/AnnalsATS.201804-261OC. PubMed PMID: 30114941.
2. Antao VC, Petsonk EL, Sokolow LZ, Wolfe AL, Pinheiro GA, Hale JM, et al. Rapidly progressive coal workers' pneumoconiosis in the United States: geographic clustering and other factors. Occup Environ Med. 2005;62(10):670-4. doi: 10.1136/oem.2004.019679. PubMed PMID: 16169911.
3. Arif AA, Paul R, Delmelle E, Owusu C, Adeyemi O. Estimating the prevalence and spatial clusters of coal workers' pneumoconiosis cases using medicare claims data, 2011-2014. Am J Ind Med. 2020;63(6):478-83. doi: 10.1002/ajim.23104. PubMed PMID: 32147857.
4. Blackley DJ, Halldin CN, Wang ML, Laney AS. Small mine size is associated with lung function abnormality and pneumoconiosis among underground coal miners in Kentucky, Virginia and West Virginia. Occup Environ Med. 2014;71(10):690-4. doi: 10.1136/oemed-2014-102224. PubMed PMID: 25052085.
5. Blackley DJ, Halldin CN, Laney AS. Continued Increase in Prevalence of Coal Workers' Pneumoconiosis in the United States, 1970-2017. Am J Public Health. 2018;108(9):1220-2. doi: 10.2105/ajph.2018.304517. PubMed PMID: 30024799.
6. Blackley DJ, Reynolds LE, Short C, Carson R, Storey E, Halldin CN, et al. Progressive Massive Fibrosis in Coal Miners From 3 Clinics in Virginia. JAMA. 2018;319(5):500-1. doi: 10.1001/jama.2017.18444. PubMed PMID: 29411024.
7. CDC. Silicosis screening in surface coal miners--Pennsylvania, 1996-1997. MMWR 2000;49(27):612-5. PubMed PMID: 10914927.
8. CDC. Pneumoconiosis prevalence among working coal miners examined in federal chest radiograph surveillance programs--United States, 1996-2002. MMWR. 2003;52(15):336-40. PubMed PMID: 12733865.
9. CDC. Advanced cases of coal workers' pneumoconiosis--two counties, Virginia, 2006. MMWR 2006;55(33):909-13. PubMed PMID: 16929235.
10. CDC. Advanced pneumoconiosis among working underground coal miners--Eastern Kentucky and Southwestern Virginia, 2006. MMWR 2007;56(26):652-5. PubMed PMID: 17615522.
11. CDC. Pneumoconiosis and advanced occupational lung disease among surface coal miners--16 states, 2010-2011. MMWR 2012;61(23):431-4. PubMed PMID: 22695382.
12. Graber JM, Cohen RA, Basanets A, Stayner LT, Kundiev Y, Conroy L, et al. Results from a Ukrainian-US collaborative study: prevalence and predictors of respiratory symptoms among Ukrainian coal miners. Am J Ind Med. 2012;55(12):1099-109. doi: 10.1002/ajim.21997. PubMed PMID: 22169933.
13. Graber JM, Harris G, Almberg KS, Rose CS, Petsonk EL, Cohen RA. Increasing Severity of Pneumoconiosis Among Younger Former US Coal Miners Working Exclusively Under Modern Dust-Control Regulations. J Occup Environ Med. 2017;59(6):e105-e11. doi: 10.1097/jom.0000000000001048. PubMed PMID: 28598937.
14. Hall NB, Blackley DJ, Halldin CN, Laney AS. Continued increase in prevalence of r-type opacities among underground coal miners in the USA. Occup Environ Med. 2019;76(7):479-81. doi: 10.1136/oemed-2019-105691. PubMed PMID: 31023786.
15. Hall NB, Halldin CN, Blackley DJ, Laney AS. Assessment of pneumoconiosis in surface coal miners after implementation of a national radiographic surveillance program, United States, 2014-2019. Am J Ind Med. 2020;63(12):1104-8. doi: 10.1002/ajim.23184.
16. Han L, Han R, Ji X, Wang T, Yang J, Yuan J, et al. Prevalence Characteristics of Coal Workers' Pneumoconiosis (CWP) in a State-Owned Mine in Eastern China. Int J Environ Res Public Health. 2015;12(7):7856-67. doi: 10.3390/ijerph120707856. PubMed PMID: 26184259.
17. Han B, Liu H, Zhai G, Wang Q, Liang J, Zhang M, et al. Estimates and Predictions of Coal Workers' Pneumoconiosis Cases among Redeployed Coal Workers of the Fuxin Mining Industry Group in China: A Historical Cohort Study. PLoS One. 2016;11(2):e0148179. doi: 10.1371/journal.pone.0148179. PubMed PMID: 26845337.
18. Kurth L, Laney AS, Blackley DJ, Halldin CN. Prevalence of spirometry-defined airflow obstruction in never-smoking working US coal miners by pneumoconiosis status. Occup Environ Med. 2020;77(4):265-7. doi: 10.1136/oemed-2019-106213. PubMed PMID: 32041810.
19. Laney AS, Attfield MD. Coal workers' pneumoconiosis and progressive massive fibrosis are increasingly more prevalent among workers in small underground coal mines in the United States. Occup Environ Med. 2010;67(6):428-31. doi: 10.1136/oem.2009.050757. PubMed PMID: 20522823.
20. Laney AS, Attfield MD. Examination of potential sources of bias in the US Coal Workers' Health Surveillance Program. Am J Public Health. 2014;104(1):165-70. doi: 10.2105/ajph.2012.301051. PubMed PMID: 23678894.

21. Laney AS, Petsonk EL, Attfield MD. Pneumoconiosis among underground bituminous coal miners in the United States: is silicosis becoming more frequent? Occup Environ Med. 2010;67(10):652-6. doi: 10.1136/oem.2009.047126. PubMed PMID: 19773275.
22. Laney AS, Petsonk EL, Hale JM, Wolfe AL, Attfield MD. Potential determinants of coal workers' pneumoconiosis, advanced pneumoconiosis, and progressive massive fibrosis among underground coal miners in the United States, 2005-2009. Am J Public Health. 2012;102 Suppl 2:S279-83. doi: 10.2105/ajph.2011.300427. PubMed PMID: 22401526.
23. Laney AS, Blackley DJ, Halldin CN. Radiographic disease progression in contemporary US coal miners with progressive massive fibrosis. Occup Environ Med. 2017;74(7):517-20. doi: 10.1136/oemed-2016-104249. PubMed PMID: 28408654.
24. Li Y, Xian W, Xu H, Sun J, Han B, Liu H. Time trends and future prediction of coal worker's pneumoconiosis in opencast coal mine in China based on the APC model. BMC Public Health. 2018;18(1):1010. doi: 10.1186/s12889-018-5937-0. PubMed PMID: 30107832.
25. Naidoo RN, Robins TG, Solomon A, White N, Franzblau A. Radiographic outcomes among South African coal miners. Int Arch Occup Environ Health. 2004;77(7):471-81. doi: 10.1007/s00420-004-0532-3. PubMed PMID: 15558299.
26. Reynolds LE, Blackley DJ, Laney AS, Halldin CN. Respiratory morbidity among U.S. coal miners in states outside of central Appalachia. Am J Ind Med. 2017;60(6):513-7. doi: 10.1002/ajim.22727. PubMed PMID: 28497853.
27. Scarisbrick D, Quinlan R. Health surveillance for coal workers' pneumoconiosis in the United Kingdom 1998–2000. Ann Occup Hyg. 2002;46(suppl_1):254-6.
28. Suarthana E, Laney AS, Storey E, Hale JM, Attfield MD. Coal workers' pneumoconiosis in the United States: regional differences 40 years after implementation of the 1969 Federal Coal Mine Health and Safety Act. Occup Environ Med. 2011;68(12):908-13. doi: 10.1136/oem.2010.063594. PubMed PMID: 21597107.
29. Tor M, Ozturk M, Altin R, Cimrin AH. Working conditions and pneumoconiosis in Turkish coal miners between 1985 and 2004: a report from Zonguldak coal basin, Turkey. Tuberk Toraks. 2010;58(3):252-60. Epub 2010/11/03. PubMed PMID: 21038135.
30. Vallyathan V, Landsittel DP, Petsonk EL, Kahn J, Parker JE, Osiowy KT, et al. The influence of dust standards on the prevalence and severity of coal worker's pneumoconiosis at autopsy in the United States of America. Arch Pathol Lab Med. 2011;135(12):1550-6. doi: 10.5858/arpa.2010-0393-OA. PubMed PMID: 22129182.
31. Wang ML, Beeckman-Wagner LA, Wolfe AL, Syamlal G, Petsonk EL. Lung-function impairment among US underground coal miners, 2005 to 2009: geographic patterns and association with coal workers' pneumoconiosis. J Occup Environ Med. 2013;55(7):846-50. doi: 10.1097/JOM.0b013e31828dc985. PubMed PMID: 23787575.
32. Attfield MD, Kuempel ED. Mortality among U.S. underground coal miners: a 23-year follow-up. Am J Ind Med. 2008;51(4):231-45. doi: 10.1002/ajim.20560. PubMed PMID: 18247381.
33. Beggs JA, Slavova S, Bunn TL. Patterns of pneumoconiosis mortality in Kentucky: Analysis of death certificate data. Am J Ind Med. 2015;58(10):1075-82. doi: 10.1002/ajim.22511. PubMed PMID: 26374490.
34. Bell JL, Mazurek JM. Trends in Pneumoconiosis Deaths - United States, 1999-2018. MMWR Morb Mortal Wkly Rep. 2020;69(23):693-8. doi: 10.15585/mmwr.mm6923a1. PubMed PMID: 32525855.
35. CDC. Coal Workers' Pneumoconiosis-Related Years of Potential Life Lost Before Age 65 Years—United States, 1968-2006. JAMA. 2010;303(16):1591-3.
36. Coggon D, Harris EC, Brown T, Rice S, Palmer KT. Work-related mortality in England and Wales, 1979-2000. Occup Environ Med. 2010;67(12):816-22. doi: 10.1136/oem.2009.052670. PubMed PMID: 20573846.
37. Graber JM, Stayner LT, Cohen RA, Conroy LM, Attfield MD. Respiratory disease mortality among US coal miners; results after 37 years of follow-up. Occup Environ Med. 2014;71(1):30-9. doi: 10.1136/oemed-2013-101597. PubMed PMID: 24186945.
38. Miller BG, MacCalman L. Cause-specific mortality in British coal workers and exposure to respirable dust and quartz. Occup Environ Med. 2010;67(4):270-6. doi: 10.1136/oem.2009.046151. PubMed PMID: 19819863.
39. Smith DR, Leggat PA. 24 years of pneumoconiosis mortality surveillance in Australia. J Occup Health. 2006;48(5):309-13. doi: 10.1539/joh.48.309. PubMed PMID: 17053296.
40. Han L, Gao Q, Yang J, Wu Q, Zhu B, Zhang H, et al. Survival Analysis of Coal Workers' Pneumoconiosis (CWP) Patients in a State-Owned Mine in the East of China from 1963 to 2014. Int J Environ Res Public Health. 2017;14(5). doi: 10.3390/ijerph14050489. PubMed PMID: 28481235.
