# Supplementary material for: A systematic review and meta-analysis on international studies of prevalence, mortality and survival due to coal mine dust lung disease
Source: PLoS One. 2021 Aug 3;16(8):e0255617. doi: 10.1371/journal.pone.0255617 (PMC8330946; doi:10.1371/journal.pone.0255617)

S4 Table Sensitivity analyses for the influence of individual studies on the pooled prevalence estimates for coal workers pneumoconiosis (CWP) in the United States. Included meta-analyses are for coal workers pneumoconiosis by disease severity (progressive massive fibrosis, advanced coal workers pneumoconiosis), mine type (surface), region (Central Appalachia versus overall United States) and r-type opacities. Each plot shows the calculated prevalence estimates with the named study omitted.

Progressive massive fibrosis

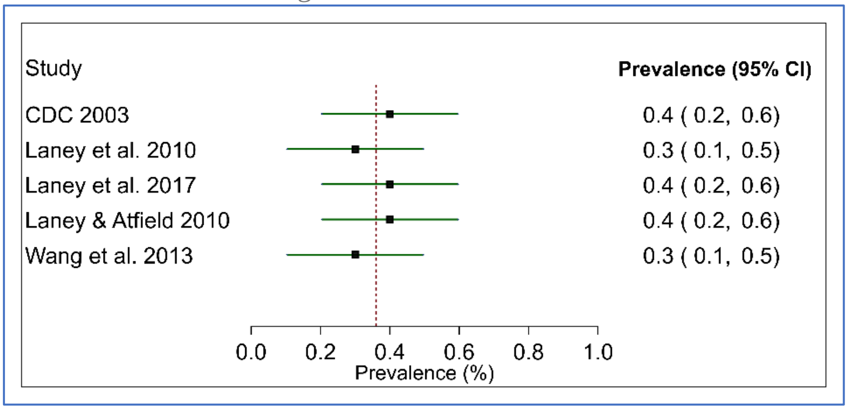

Advanced CWP

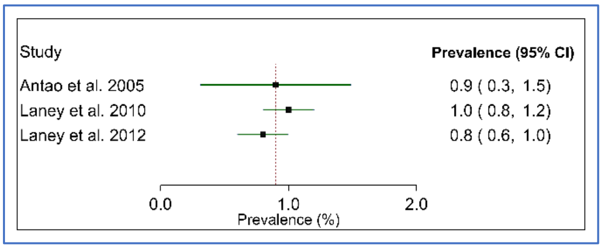

Surface mines-CWP

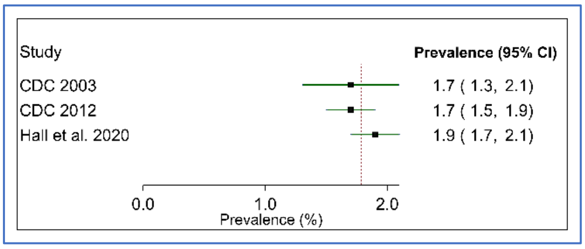

r-type opacities

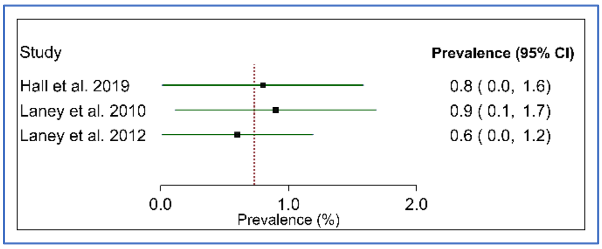

CWP (Central Appalachia, *includes states Kentucky, Virginia, and West Virginia in the USA*)

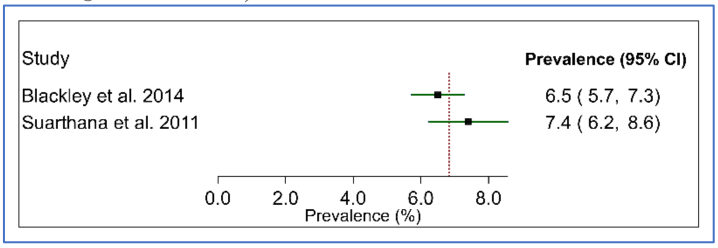

CWP (National, all USA)

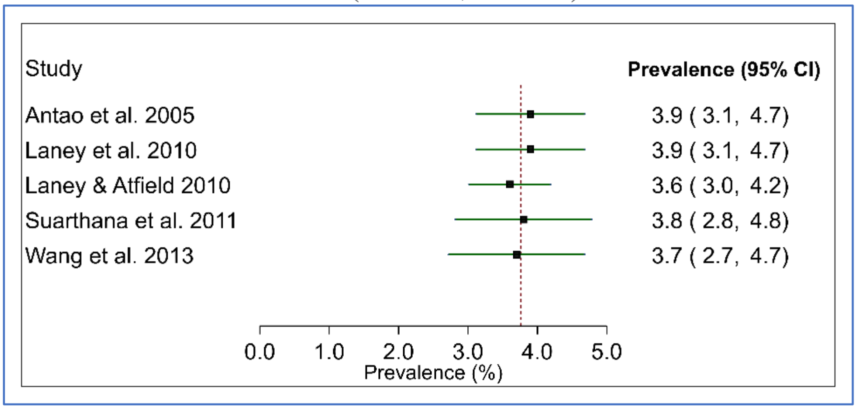

Supplement: S4 Table — Included meta-analyses are for coal workers pneumoconiosis (CWP) by disease severity (progressive massive fibrosis, advanced coal workers pneumoconiosis), mine type (surface), region (Central Appalachia versus overall United States) and r-type opacities. (PDF) [file pone.0255617.s005.pdf]
