# Supplementary material for: A systematic review and meta-analysis on international studies of prevalence, mortality and survival due to coal mine dust lung disease
Source: PLoS One. 2021 Aug 3;16(8):e0255617. doi: 10.1371/journal.pone.0255617 (PMC8330946; doi:10.1371/journal.pone.0255617)

S5 Table Forest plot from meta-analysis for prevalence of coal workers pneumoconiosis (top) and progressive massive fibrosis (bottom) in the United States by study time period (1990s versus 2000s).

Coal workers pneumoconiosis

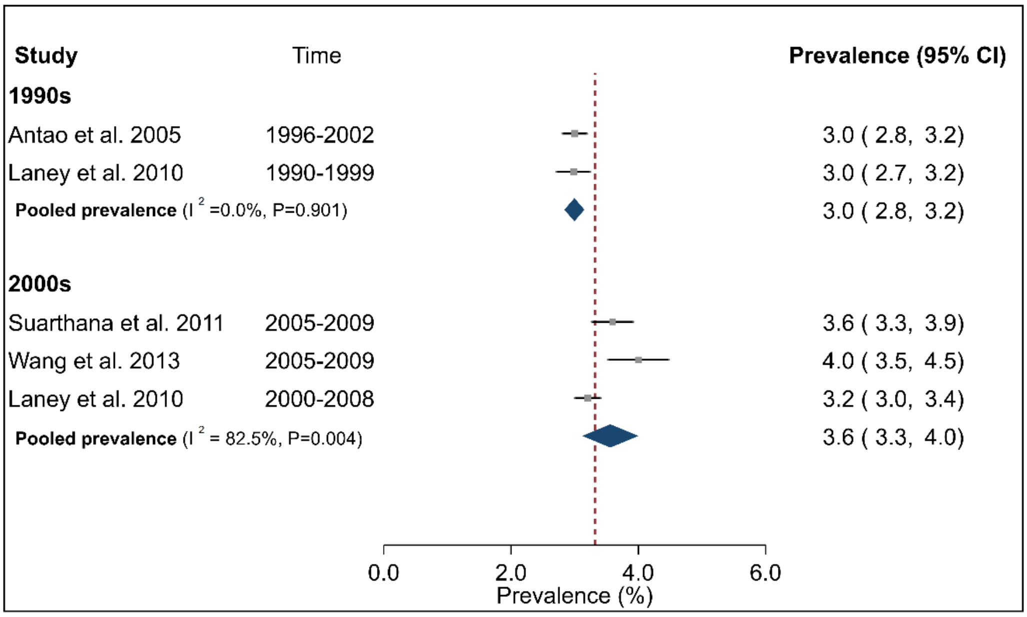

Progressive massive fibrosis

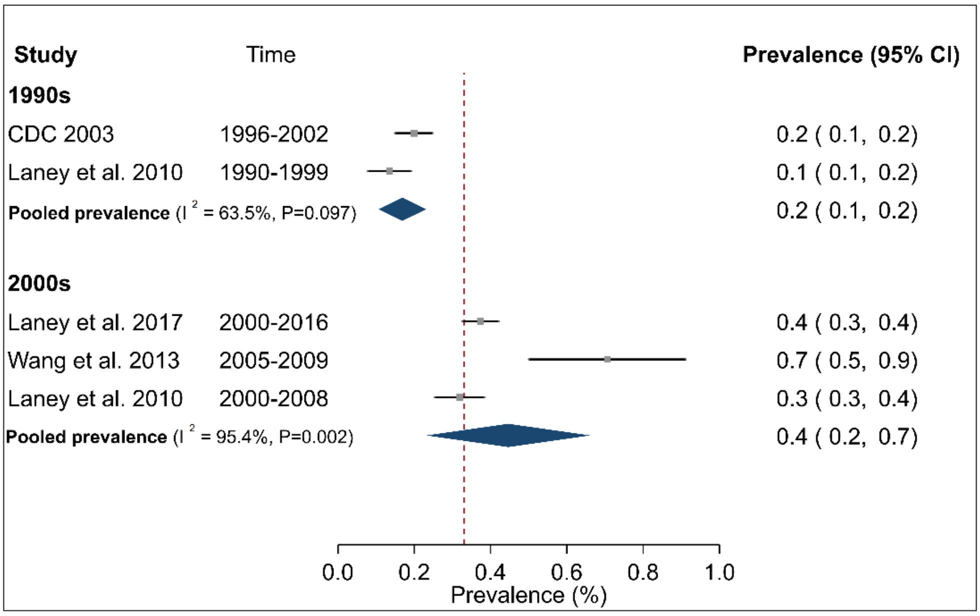

Supplement: S5 Table — (PDF) [file pone.0255617.s006.pdf]
