# Supplementary figures and images for: A systematic review and meta-analysis on international studies of prevalence, mortality and survival due to coal mine dust lung disease
Source: PLoS One. 2021 Aug 3;16(8):e0255617. doi: 10.1371/journal.pone.0255617 (PMC8330946; doi:10.1371/journal.pone.0255617)

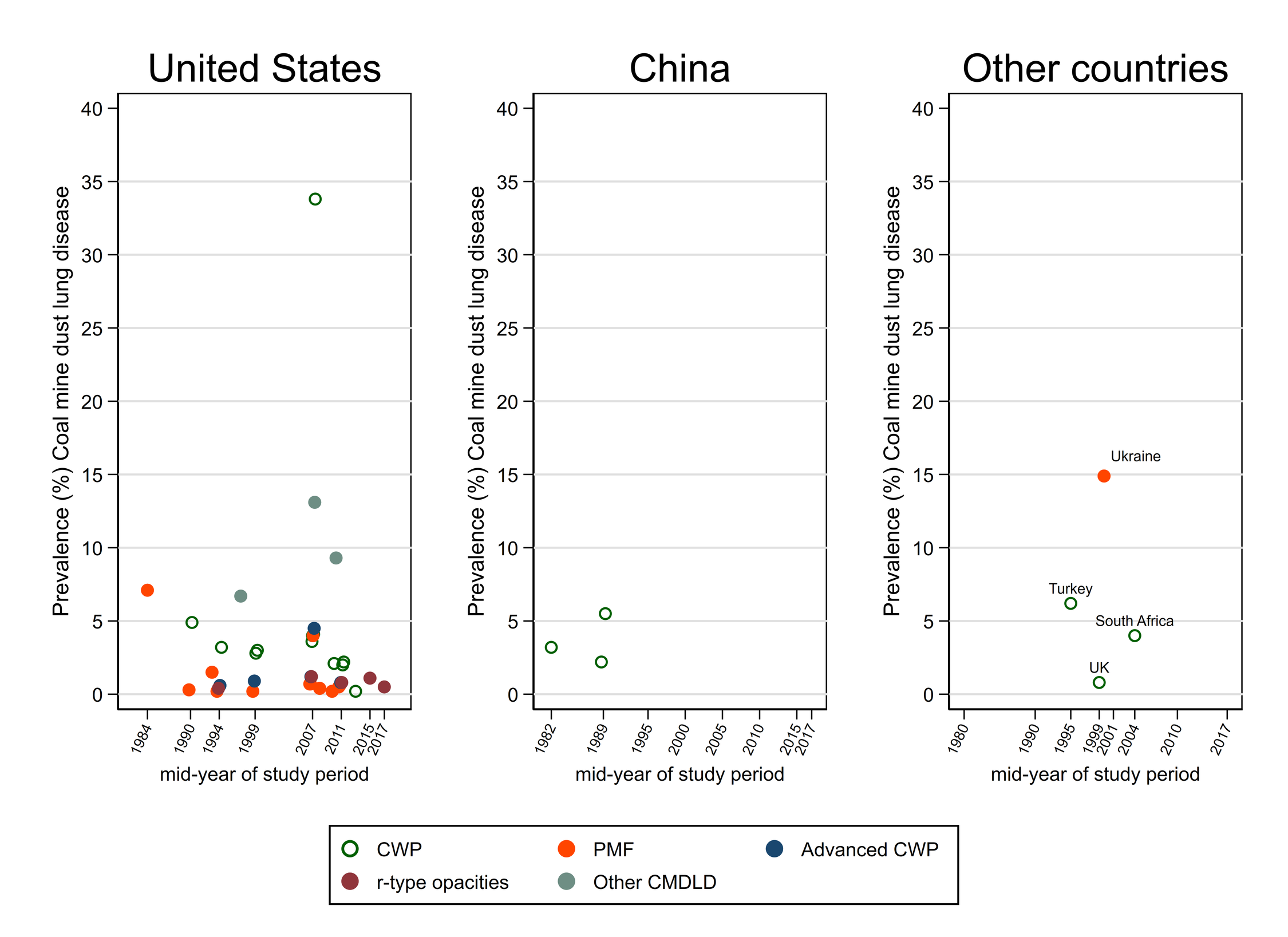

Supplement: S1 Fig — Abbreviations are USA United States, UK United Kingdom, CMDLD coal mine dust lung disease, CWP coal workers pneumoconiosis, PMF progressive massive fibrosis, Advanced CWP advanced coal workers pneumoconiosis. Other CMDLD includes silicosis, lung function abnormality and chronic obstructive pulmonary disease. Other countries are UK, Turkey, South Africa and Ukraine. Within each country, studies are shown by mid-year of the study period. (TIF) [file pone.0255617.s007.tif]

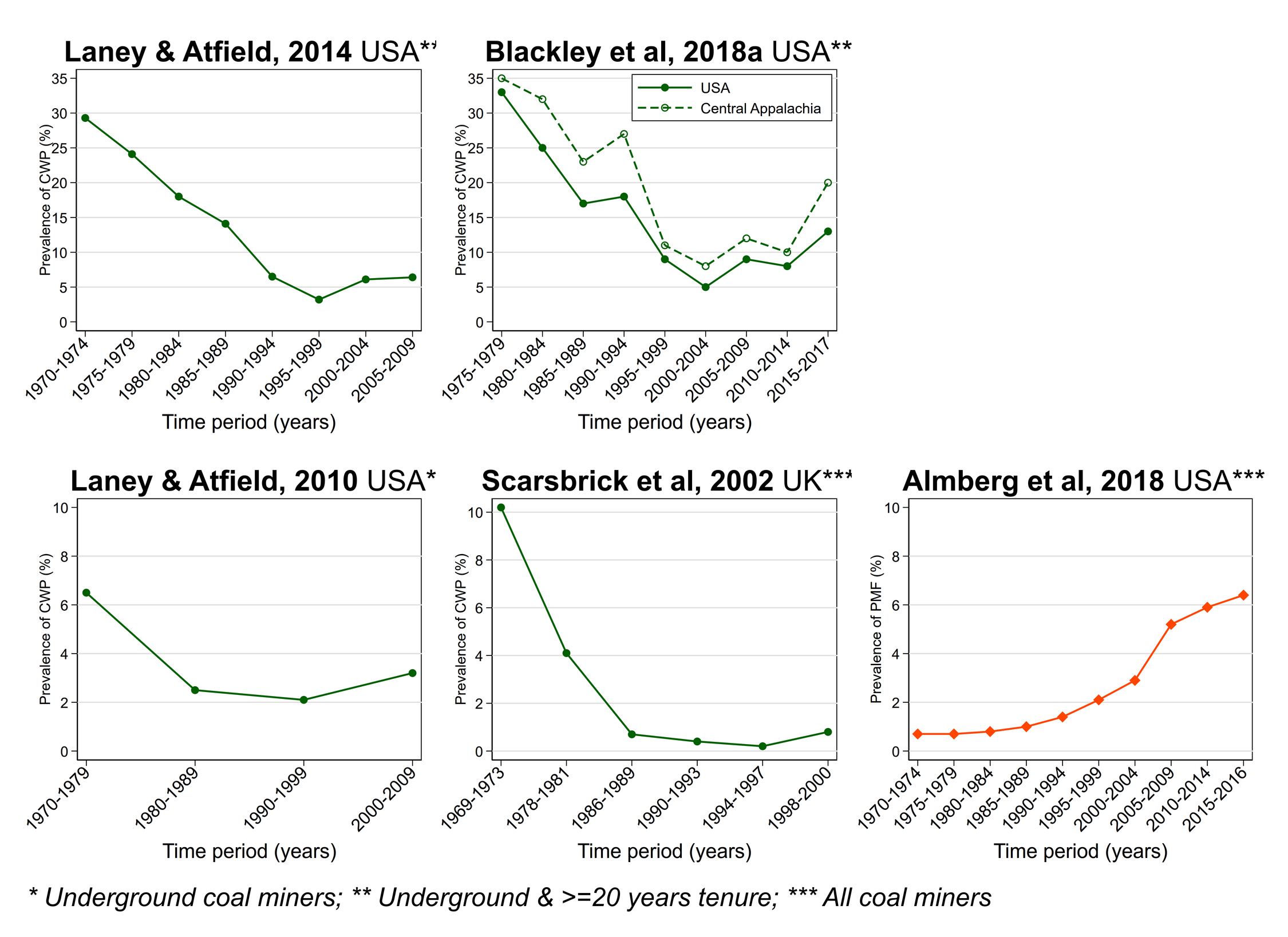

Supplement: S2 Fig — Data was sourced from Laney & Atfield 2010 [32]; Laney & Atfield 2014 [33] Blackley et al 2018a [22], Almberg et al 2018 [15] and Scarsbrick et al 2002 [50] in the systematic review. Please note that the time periods reported is limited to those reported by included studies. Trends for CWP prevalence are shown in green and for PMF prevalence in red. Abbreviations are USA United States, UK United Kingdom, CWP coal workers pneumoconiosis, PMF progressive massive fibrosis. Central Appalachia includes the states of Kentucky, Virginia, and West Virginia in USA. (TIF) [file pone.0255617.s008.tif]

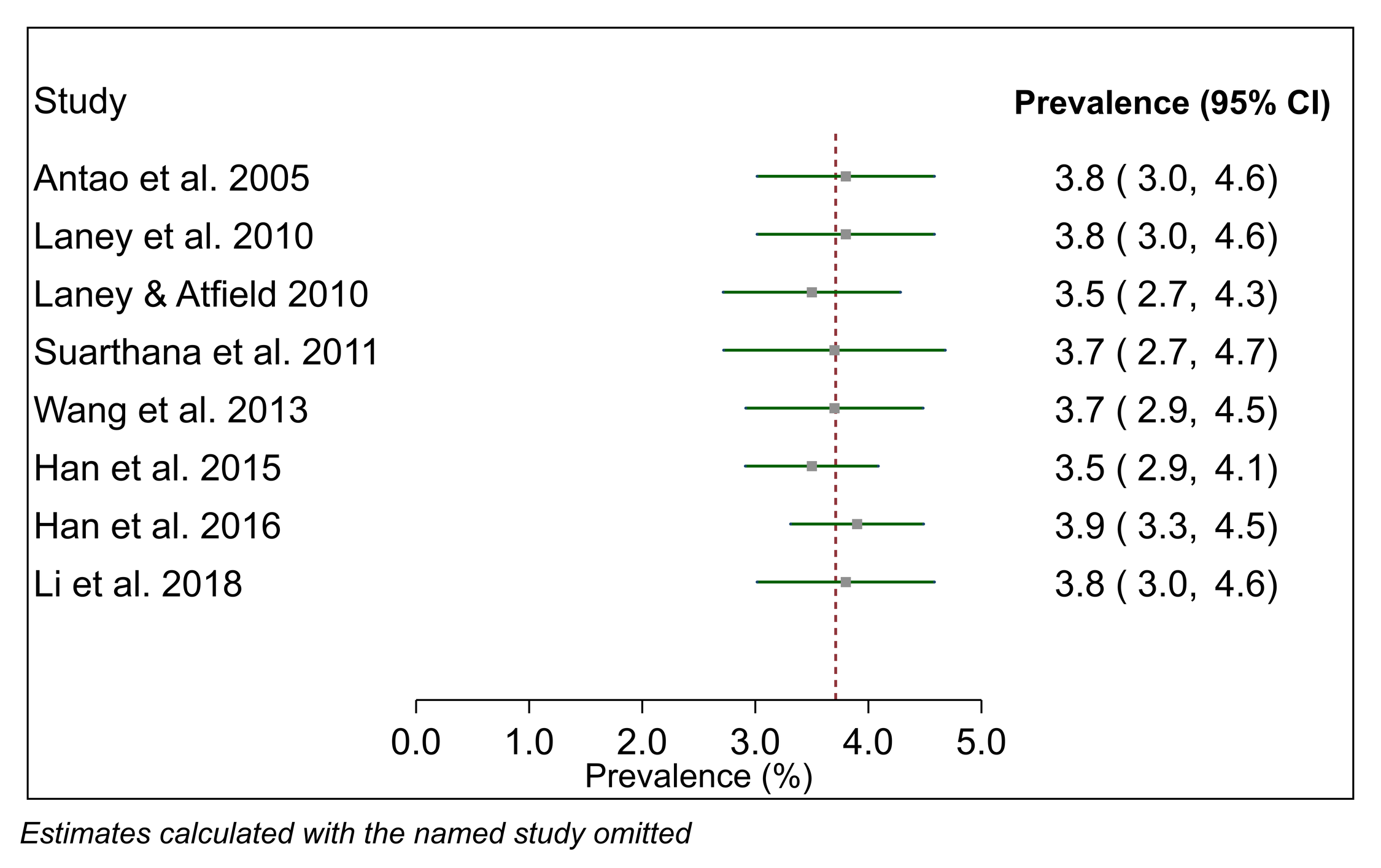

Supplement: S3 Fig — (TIF) [file pone.0255617.s009.tif]

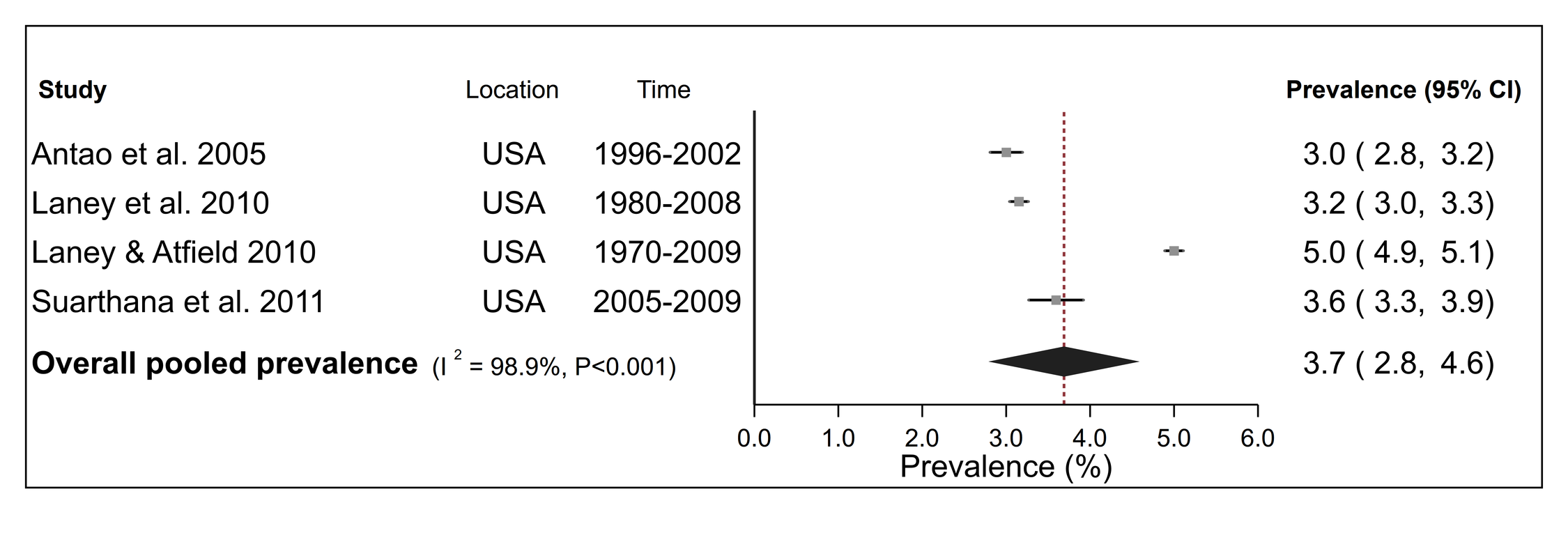

Supplement: S4 Fig — (TIF) [file pone.0255617.s010.tif]

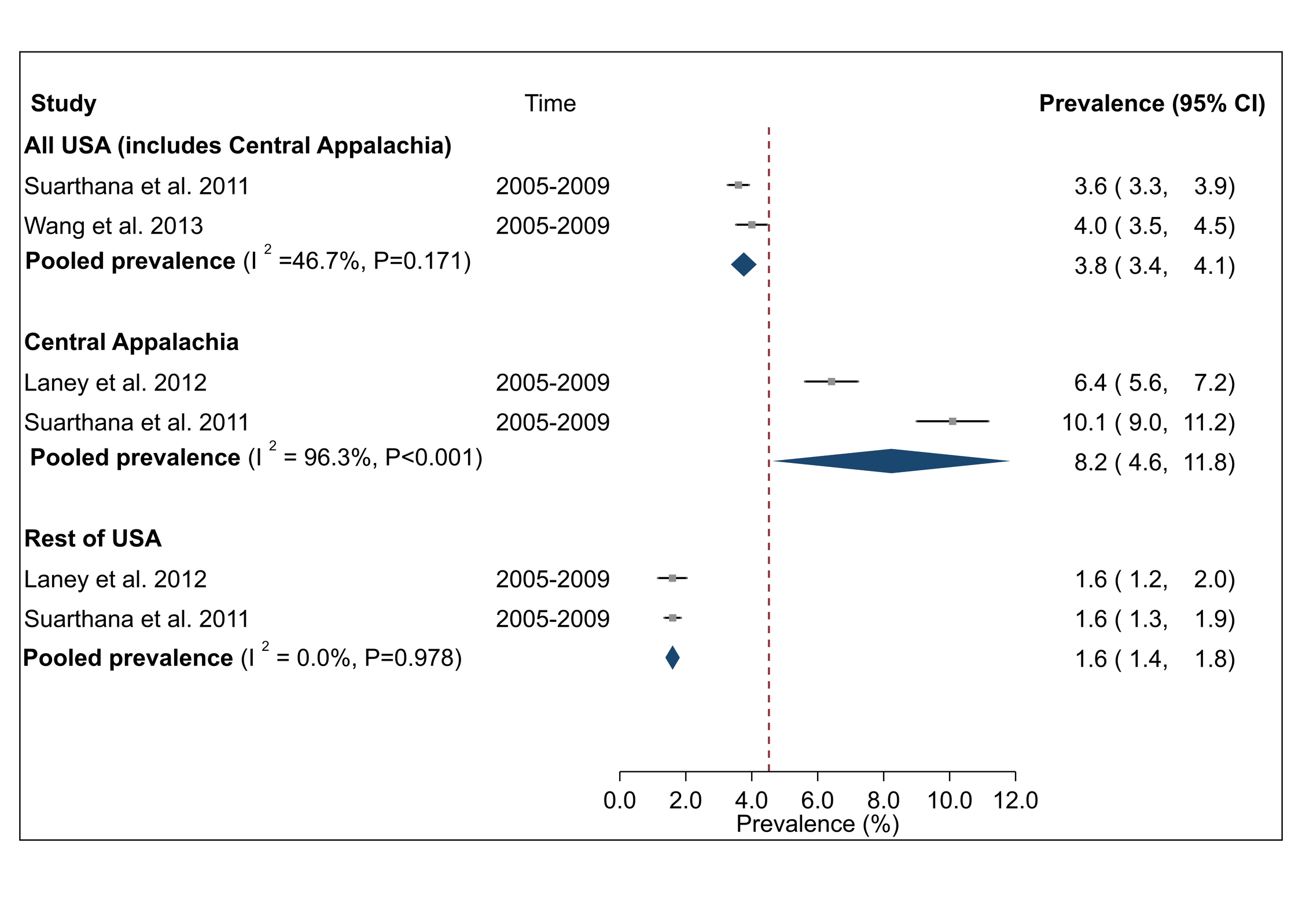

Supplement: S5 Fig — The term Central Appalachia refers to the region covered by states of Kentucky, Virginia, and West Virginia. (TIF) [file pone.0255617.s011.tif]
